# Quantitative CT Metrics for the Prediction of Therapeutic Effect in Asthma

**DOI:** 10.3390/jcm12020639

**Published:** 2023-01-13

**Authors:** Donghai Ma, Honglei Shi, Cuiyan Tan, Wei Zou, Fengfei Sun, Kongqiu Wang, Qianqian Lei, Xiaobin Zheng, Yuanyuan Zhong, Changli Tu, Meizhu Chen, Yiying Huang, Zhenguo Wang, Jian Wu, Yingjian Liang, Jing Liu

**Affiliations:** 1 Department of Respiratory and Critical Care Medicine, The Fifth Affiliated Hospital of Sun Yat-sen University, Zhuhai 519000, China; 2 Guangdong Provincial Key Laboratory of Biomedical Imaging and Guangdong Provincial Engineering Research Center of Molecular Imaging, The Fifth Affiliated Hospital Sun Yat-sen University, Zhuhai 519000, China

**Keywords:** asthma, spirometry, impulse oscillometry, budesonide/formoterol, phenotypes

## Abstract

Background: Few studies have explored the correlation between asthma medication and features on HRCT images. We aim to analyse the differences and temporal changes of lung function and airway resistance in asthma with diverse HRCT phenotypes in a short period after inhalation of budesonide/formoterol. Method: This observational study recruited 55 adult patients with varying severities of asthma. We performed detailed airway metrics measurements of chest CT scans, such as airway wall thickness (WT), wall area percentage (WA%), wall thickness percentage (T/OR), and airways with an inner perimeter of 10 mm (Pi10). The effect of lung structural features on asthma medication response was explored according to the WA% and T/OR twelve hours post-drug administration. Using multivariable regression models, we then assessed the influence of WA% on lung function. Results: WA% (*p* < 0.001) and T/OR (*p* < 0.001) significantly increased in asthma than in healthy control subjects. Compared to mild asthma, airway walls were further thickened (WA%, *p* = 0.023; T/OR: *p* = 0.029) and associated with lumen narrowing (Pi10, *p* = 0.055) in moderate to severe asthma. WA% and T/OR correlated well with lung function (FEV1, FVC, MMEF, and PEF) and airway resistance (R5, R20, Rp, and Fres). Regression analysis showed that MEF25 decreased with increasing age and WA% (R^2^ = 0.58, *p* < 0.001). Patients with thickened airway walls experienced a maximal increase in FVC, FEV1, and PEF at 2 h (*p* < 0.001) and a maximal decrease of R5, Z5, and Rp at 2 h (*p* < 0.001) in those with a thickened airway pattern. Conclusions: Asthma patients with different bronchial wall thicknesses exhibited variable lung function changes. Specifically, patients with thick airway wall patterns were more sensitive to inhaled budesonide in the short term.

## 1. Introduction

Asthma is one of the most common respiratory diseases, with diverse phenotypes and underlying pathogenetic mechanisms [1], affecting over 300 million adults worldwide [2]. The entire bronchial tree is involved in the pathophysiology of asthma, comprising both the large and small airways. Due to chronic inflammation, several changes such as increased airway resistance, narrowing, and remodelling are also exhibited [2,3].

Quantitative analysis of chest CT images is becoming increasingly valuable for evaluating airway changes in subjects with chronic airway disease [4], since manually measuring airways on clinical CT images is a complicated process. Some new techniques have been developed and validated to facilitate airway metrics measurements. The Chest Imaging Platform extension for 3D Slicer, funded by the National Heart, Lung, and Blood Institute of the National Institutes of Health, is free, open-source software available on multiple operating systems and seems less affected by different reconstruction kernels and radiation doses [5]. The method has been proven effective in previous studies on chronic airway diseases, not limited to COPD and asthma [6,7]. Using this AI software, a series of airway metrics were automatically generated, including the number of segmental and subsegmental bronchi, luminal diameter, airway wall thickness (WT), wall area percentage (WA%), and lumen diameter/lumen perimeters [8].

Impulse oscillometry is a non-invasive method of measuring lung function by exerting external pressure signals at different frequencies on normal tidal breathing. It is usually performed in paediatric or geriatric patients due to its ease of use and the need for less patient cooperation. It is now being implemented in many pulmonary function laboratories to evaluate lung function, mainly used in evaluating small airway changes [9]. Combined with traditional spirometry, IOS may increase the clinical recognition of some airway diseases in the early stage. In fact, we have proved the usefulness of IOS in evaluating and predicting disease severity in bronchiectasis [10].

Previous studies suggest that airway imaging features such as airway remodelling and airway walls of patients with asthma are significantly thicker than those of healthy controls [11]. In addition, it has been shown that greater airway wall thickness is associated with more severe airflow obstruction in asthma [12]. Symbicort is a long-term maintenance medicine containing budesonide, a corticosteroid, and formoterol, a rapid and long-acting beta2-agonist, used twice daily to treat asthma in adults. The drug functions fifteen minutes after inhalation, providing a rapid, clinically effective bronchodilator response and relieving lung inflammation [13,14]. However, to our knowledge, no previous studies have assessed lung function during serial follow-ups in a predefined period, especially in exploring the medication effects and the correlation with airway measurement metrics.

In summary, the influence of airway imaging metrics on the therapeutic effect remains unclear. In this study, we aim to analyse the baseline lung function and airway resistance in asthma patients with different HRCT phenotypes and to observe dynamic changes in each group at 12 hours’ follow-up after inhalation of budesonide/formoterol.

## 2. Materials and Methods

### 2.1. Patient Recruitment

This single-centred, observational study recruited 55 stable asthma patients and 51 healthy controls between November 2018 and January 2020 from the Fifth Affiliated Hospital of Sun Yat-Sen University. To be included in this study, subjects had to be aged over 18 years and agreed to perform repetitive pulmonary function tests. Adults enrolled in this study were those with a confirmed asthma diagnosis in a relatively stable condition without acute asthma exacerbation in the previous four weeks. Asthma patients with any of the following co-morbidities were excluded: Churg-Strauss syndrome, interstitial pneumonia, bronchiectasis, tuberculosis, lung cancer, autoimmune disease, heart failure, and renal failure. The healthy controls included staff members and people who underwent health check-ups, with no respiratory symptoms in their past illness history and a normal chest CT scan. We obtained written informed consent from all participants before testing procedures. The institutional ethics committee of the Fifth Affiliated Hospital of Sun Yat-sen University approved the study project.

### 2.2. Diagnosis of Asthma 

The diagnosis of asthma is based mainly on clinical symptoms together with airway obstruction and reversible airflow restriction following national and international guidelines [2,15,16]. Lung function and airway resistance was evaluated first to determine whether the patient presented an obstructive or restrictive pattern. Then the following objective test was made to confirm the diagnosis: (1) Positive bronchodilator responsiveness: an increase in FEV1 of >12% and >200 mL from the baseline level ten minutes after 200 mcg of salbutamol or (2) an increase in FEV1 of >12% and >200 mL (or PEF by >20%) from the baseline after four weeks of treatment, outside any respiratory infections; (3) Positive bronchial challenge test: a decrease in FEV1 of ≥20% from the baseline with standard doses of methacholine. Asthma patients were staged following the GINA recommendations as mild, moderate, and severe [1].

### 2.3. Performance of Chest CT

Chest CT was performed in all patients and healthy controls as per the method applied in our previous study [17]. In brief, all subjects underwent HRCT (SOMATOM Definition Flash Siemens; Erlangen, Germany) scans without contrast in the craniocaudal direction while placed in the supine position during end-inspiration. The 16-row multi-probe CT scanner was used for CT examination, and the following parameters were adopted: 120 kVp, 150 mA, 1.5 mm collimation, 1.35:1 pitch, and sharp core (B80f). The images were then reconstructed at 1.0 mm slice thickness, with 1 mm increment, 512 mm × 512 mm matrix. 

### 2.4. Quantitative CT Analysis of Airways

**Figure 1:**
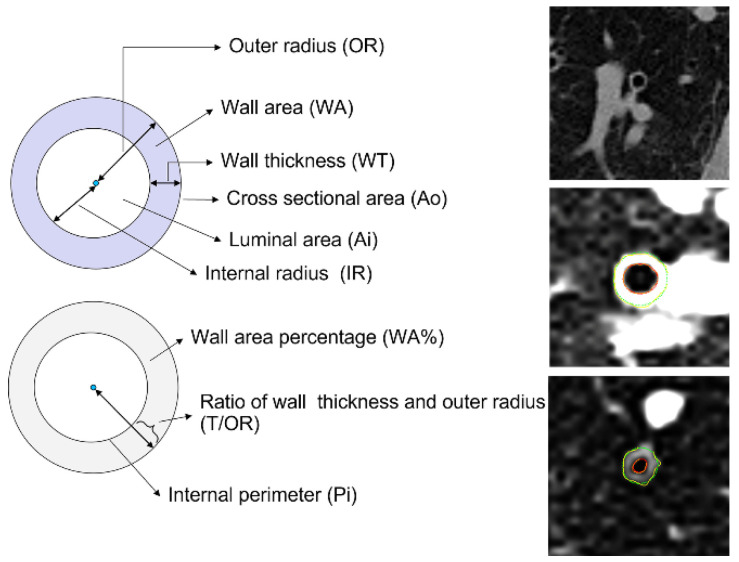
Indices of airway wall thickness (**left part**) and representative HRCT scans (**right part**).

For all scans, bronchial wall metrics were obtained in cross-sectionally reformatted images for all segmental and subsegmental bronchi. The images of each bronchus examined were approximately perpendicular to the bronchial axis (Figure 1). We used the AI tool of the Chest Imaging Platform that could automatically extract airway centrelines. The airway bounder line is detectable when no adjacent tissue of similar CT density is present and should be considered acceptable when at least 25% of the circumference is detected at a specific location. The software can provide the cross-sectional measurement of airways characteristics, including the number of segmental airways counted, airways with an internal perimeter of 10 mm (Pi10), inner and outer airway diameter, the absolute value of wall thickness, and inner and outer area. In addition, normalised airway metrics such as the wall area percentage (WA%), square root of WA, and the ratio of airway wall thickness to the outer radius (T/OR) were automatically generated. The images were viewed at a window level of –450 HU and a window width of 1500 HU.

### 2.5. Stratification of Asthma according to HRCT Metrics

In order to study the drug responsiveness in patients with different airway wall thicknesses, we selected the two normalised airway thickness indicators, the WA% and T/OR ratio, and classified the patients into a low-thickness group and high-thickness groups, with the cut-off value being the median of each indicator.

### 2.6. Pulmonary Function Test

Patients were asked to refrain from taking inhaled bronchodilators, at least 8 h in the case of short-term, 24 h for twice-daily ICS/LABA, and 36 h for once-daily ICS/LABA, according to the summary of product characteristics. The IOS test was performed first as a no-force-dependent procedure using the Master Screen/Sentry Suite^®^ IOS system (CareFusion Co., San Diego, CA, USA). We measured values of airway impedance at 5 Hz (Z5), airway resistance (R5; R20), reactance at 5 Hz (X5), and the resonant frequency (Fres). Asthma patients were given two puffs of Symbicort (160 mcg/4.5 mcg) orally after the performance of baseline pulmonary function testing. Spirometry, body plethysmography, and IOS test were repeated at the following time points: 30 min, 1st hour, 2nd hour, 4th hour, 8th hour, and 12th hour. Pulmonary function measurements, including forced vital capacity (FVC), forced expiratory volume in one second (FEV1), peak expiratory flow (PEF), and maximal expiratory flow at 75% to 25% of FVC (MEF) were documented. All procedures were performed following the standardised guidelines of the European Respiratory Society (ERS) and American Thoracic Society (ATS) [18].

### 2.7. Statistical Analysis

Categorical variables were presented as frequencies and percentages, and the statistical differences were analysed using the Chi-square test (or Fisher’s exact test when appropriate). Descriptive summaries of demographical variables and baseline lung function measurements included means (±SD) for normally distributed variables and medians with interquartile range (IQR) for other variables. We used the Shapiro-Wilk test to estimate the normality of study variables. Spearman’s rank correlation coefficient was used to analyse the correlation between airway indexes, lung function, and airway resistance. A multiple linear regression model was used to investigate the influence of WA%, T/OR, and age on FEV1 and other lung function parameters. To analyse data with repeated measurements on individuals at sequencing time points, a two-way repeated measures ANOVA was used, followed by Bonferroni’s post hoc test. Statistical significance was defined as *p* < 0.05. All statistical analysis was performed using Stata version 15.1 (StataCorp; College Station, TX, USA) and GraphPad Prism version 8.0 (San Diego, CA, USA).

## 3. Results

### 3.1. Characteristics of Participants

Of the 55 asthma subjects, thirty-seven (67%) patients presented mild disease. Baseline characteristics are summarised in Table 1. The group of asthma patients with moderate to severe disease was relative older (mean age 49.7 years vs. 40.3 years, *p* = 0.006), more likely to be currently smoking (22% vs. 11%, *p* = 0.045), and associated with the female gender (83% vs. 41%, *p* = 0.003) and increased blood eosinophils (*p* = 0.015). 

Regarding the impulse oscillometry metrics, increased airway resistance, i.e., the total airway resistance R5, the peripheral airway resistance Rp, the middle to small airway resistance (R5-R20), and airway impedance (Z5) were observed in patients with moderate to severe disease. In contrast, the large airway (R20) and the central airway resistance (Rc) did not yield a significant difference compared to subjects with mild disease. The resonant frequency (Fres) was significantly increased in moderate to severe asthma (19.5 Hz vs. 13.7 Hz, *p* < 0.001). Similarly, worse lung function (FEV1, FVC, FEV %predicted, and FVC % predicted), greater airway obstruction (FEV1/FVC, and FEV1% predicted), and a larger proportion of gas trapping (RV% predicted, TLC% predicted, and RV/TLC ratio) were observed in the relatively severe asthma group. The mean value of peak expiratory flow was significantly decreased in moderate to severe asthma (see Table 1).

### 3.2. Asthma Patients Present Narrowing Lumen and Increased Airway Wall Thickness 

The findings of computed tomography (CT) metrics in asthma and healthy controls are shown in Table 2. The number of countable airways was significantly higher in the asthmatic group than in healthy controls (median 19.0 vs. 15.5, *p* = 0.002). The median internal radius and outer radius of airways decreased in asthma patients compared to the controls (IR, 1.78 mm vs. 2.07 mm, *p* < 0.001; OR, 3.63 mm vs. 3.97 mm, *p* < 0.001). Accordingly, the lumen area (Ai) and cross-sectional area (Ao) of airways in asthma patients decreased as well (Ai, 11.62 vs. 15.38, *p* < 0.001; Ao, 44.86 vs. 52.55, *p* < 0.001). However, the absolute value of wall thickness in asthma patients and healthy controls showed no statistical difference. Using adjusted parameters like T/OR and WA %, a higher grade of wall thickness was demonstrated, with an increase in T/OR (median 0.50 vs. 0.46, *p* < 0.001) and WA% (median 75.53 vs. 71.21, *p* < 0.001) in asthma patients. The internal perimeter (Pi) of airways in asthma patients decreased compared to the control group (median 11.2 mm vs. 13.0 mm, *p* < 0.001). Furthermore, the percentage of airways with a perimeter more than 10 mm was mostly observed in asthma patients (98% vs. 75%, *p* < 0.001) than in healthy controls. We further compared these radiographical features in asthma subgroups. We found that patients with moderate to severe disease presented a greater lumen narrowing and airway thickness, as expressed by T/OR (0.51 vs. 0.48, *p* = 0.029) and WA % (76.78 vs. 73.65, *p* = 0.023) (see Table 2).

### 3.3. HRCT Metrics Correlated Well with Lung Function Measured Both by Spirometry and IOS

**Figure 2:**
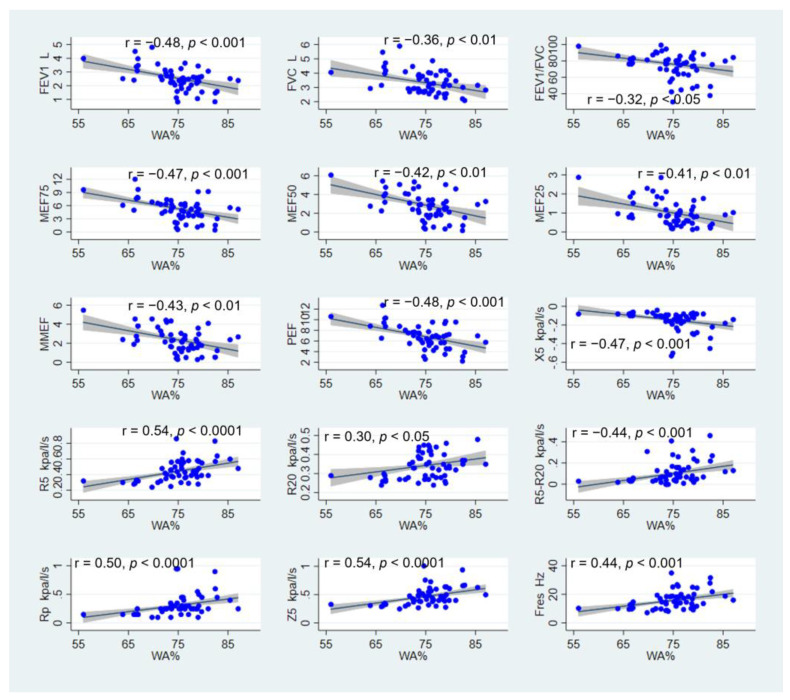
Correlation between WA% and lung function measured by spirometry and IOS.

As shown in Figure 2, WA% correlated negatively with FEV1 (r = −0.48, *p* < 0.001), FVC (r = −0.36, *p* < 0.01), ratio of FEV1/FVC (r = −0.32, *p* < 0.05), MMEF75 (r = −0.47, *p* < 0.001), MEF50 (r = 0.42, *p* < 0.01), MEF25 (r = −0.41, *p* < 0.01), MMEF (r = −0.43, *p* < 0.01), and PEF (r = −0.48, *p* < 0.001). Regarding the IOS parameters, WA% showed a positive correlation with R5 (r = 0.54, *p* < 0.0001), R20 (r = 0.30, *p* < 0.05), R5-R20 (r = 0.44, *p* < 0.001), Rp (r = 050, *p* < 0.0001), Z5 (r = 0.54, *p* < 0.0001), and Fres (r = 0.44, *p* < 0.001). An inverse correlation was found between WA% and X5 (r = −0.47, *p* < 0.001). Similarly, when considering the correlation between the T/OR ratio and lung function parameters, decreasing lung function also correlated with an increase in airway wall thickness and narrowing of the airway lumen (Figure S1). In contrast to patients with asthma, no significant correlation was noted between the wall thickness index and lung function parameters in healthy controls (Table S1).

### 3.4. Multiple Linear Regression Analysis Suggested That MEF25 Decreased with Increasing Age and WA%

**Figure 3:**
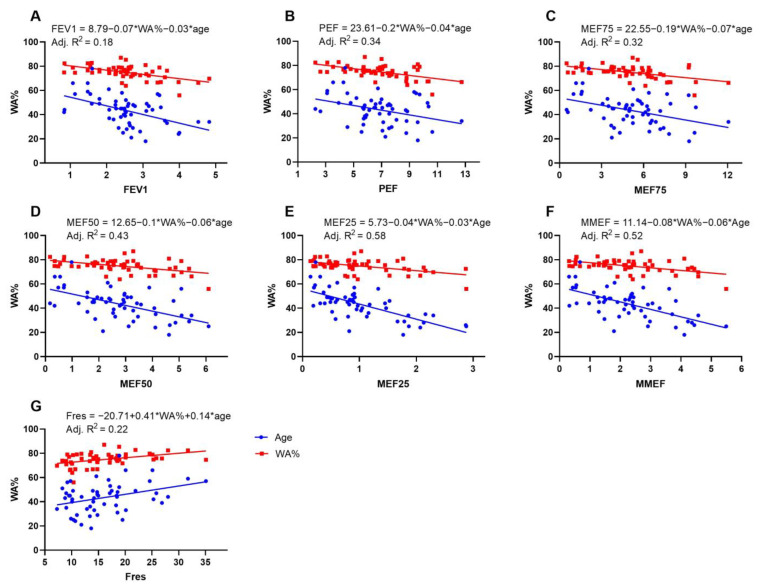
Multivariate linear regression. The blue and red symbols represent age and WA%, respectively. (**A**) FEV1, forced expiratory volume in one second; (**B**) PEF, peak expiratory flow; (**C**) MEF75, maximal expiratory flow at 75% of the FVC; (**D**) MEF50, maximal expiratory flow at 50% of the FVC; (**E**) MEF25, maximal expiratory flow at 25% of the FVC; (**F**) MMEF, maximal mid-expiratory flow; (**G**) Fres, resonance frequency.

We used multiple linear regression to predict spirometry and IOS lung function parameters based on age, WA%, and T/OR ratio. A linear relationship between independent and dependent variables is determined by plotting fractional regression scatter plots and scatter plots of residuals versus predicted values. As WA% and T/OR showed multicollinearity confirmed by the regression tolerance, only WA% was chosen as the privileged independent variable. The multiple linear regression model showed that MEF25 decreased with increasing age and WA% (F = 19.70, *p* < 0.001, R^2^ = 0.58). The effects of the two independent variables (WA% and age) included in the model on MEF25 were all statistically significant (*p* < 0.01), and the specific results are shown in Figure 3. Similarly, we found that PEF, MEF75, MEF50, FEV1, and MMEF decreased with increasing age and WA%, but not FVC, FEV1/FVC, and RV/TLC. Fres tended to increase with increasing age and WA% (F = 8.54, *p* < 0.001, R^2^ = 0.22) (Figure 3).

### 3.5. Patients with Thickened Airway Walls Experienced Greater Response after Inhalation of Budesonide/Formoterol

**Figure 4:**
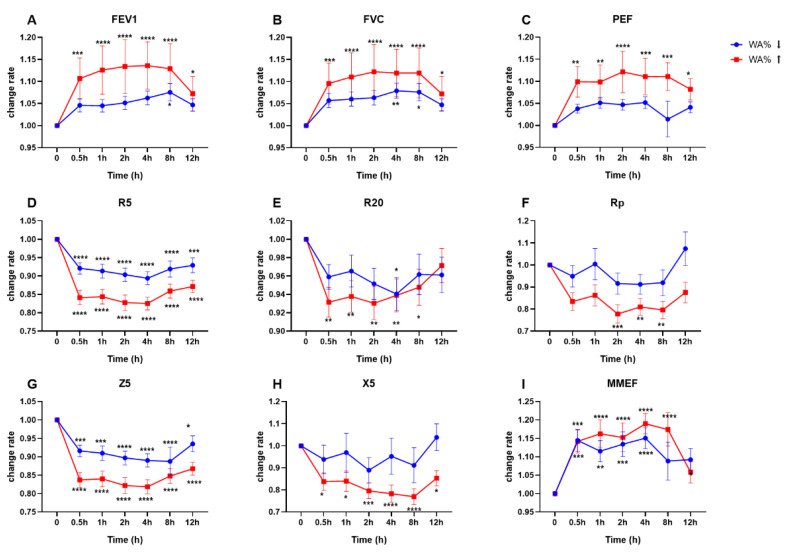
Temporal changes of lung function based on WA%. (**A**) FEV1, forced expiratory volume in one second; (**B**) FVC, forced vital capacity; (**C**) PEF, peak expiratory flow; (**D**) R5, airway resistance at 5 Hz; (**E**) R20, airway resistance at 20 Hz; (**F**) Rp, peripheral resistance; (**G**) Z5, impedance value at 5 Hz; (**H**) X5, reactance at 5 Hz; (**I**) MMEF, maximal mid-expiratory flow. The blue symbol means subject with relative non-thickened airways, and the red colour represents cohort with relative thickened airways. The dichotomisation threshold was generated based on the median value of WA%. * *p* < 0.05, ** *p* < 0.01, *** *p* < 0.001, **** *p* < 0.0001. Comparisons were made between the specific timepoint and baseline lung function.

According to the degree of airway wall thickness, asthma patients were stratified into groups with high and low WA% (or T/OR). It was worth noting that the curves of FEV1, FVC, PEF, and MMEF showed significant changes half an hour after inhalation of budesonide/formoterol in the high WA% group, and this effect lasted until 12 hours’ follow-up (Figure 4). In contrast, there was no statistically significant change in FEV1 and PEF in the low WA% group. FVC significantly improved only at the 4th and 8th hours post-drug medication in patients with low WA%. Regarding IOS parameters, both Z5 and R5 changed significantly half an hour after medication, which did not seem to be significantly related to the airway wall thickness. R20, which generally represents a large airway resistance, tended to decrease in the high WA% group at 30 min post medication and maintained up to the 8th hour. X5, the airway reactance at 5 Hz, was sensitive to budesonide/formoterol in the high WA% group but did not present any significant change in patients with low WA% (Figure 4). Changes in lung function parameters based on the T/OR ratio showed similar trends (Figure S2).

## 4. Discussion

The main new finding of this study is the acute response in lung function and airway resistance to budesonide/formoterol based on quantitative CT metrics. Our results suggested that asthma patients with relatively thickened airway walls showed greater improvement in FEV1, FVC, and PEF half an hour after drug inhalation. This effect lasted up to twelve hours of follow-up. Additionally, this study verified the correlation between HRCT metrics and lung function, showing that the thickness of the airway wall in asthma patients changes with the severity of the disease. We also approved the applicability of the IOS in evaluating airway structure changes and medication effects in asthmatic patients.

The most remarkable finding of the present study is that asthmatic patients with thickened airway walls experienced a greater improvement in FEV1, FVC, and PEF half an hour after administration of budesonide/formoterol. In contrast, another group of asthma patients with thinner airway walls seemed less sensitive to inhaled medication. Successfully phenotyping asthma is essential for precise and personalised medicine [1]. Taking advantage of the radiographical characteristics of HRCT imaging, we can apparently distinguish two asthma subtypes with different drug responsiveness. To explain why asthma patients with diverse airway wall thickness show different drug responsiveness, we should focus on the pathology of the disease itself. As known, in asthma, chronic bronchial inflammation and airway remodelling may increase the bronchial wall thickness [19], which is the main morphological feature of stable asthma assessed using post-mortem specimens [20].

**Figure 5:**
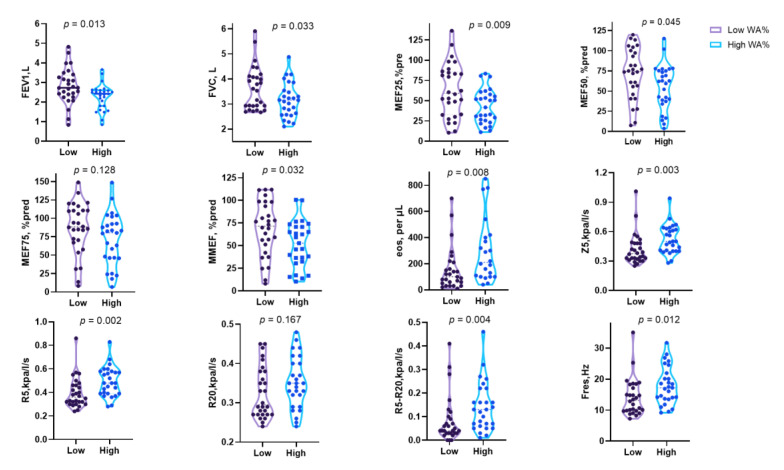
Spirometry and lung airway resistance parameters according to the airway wall thickness stratified by WA%. Data expressed as median and interquartile range.

**Figure 6:**
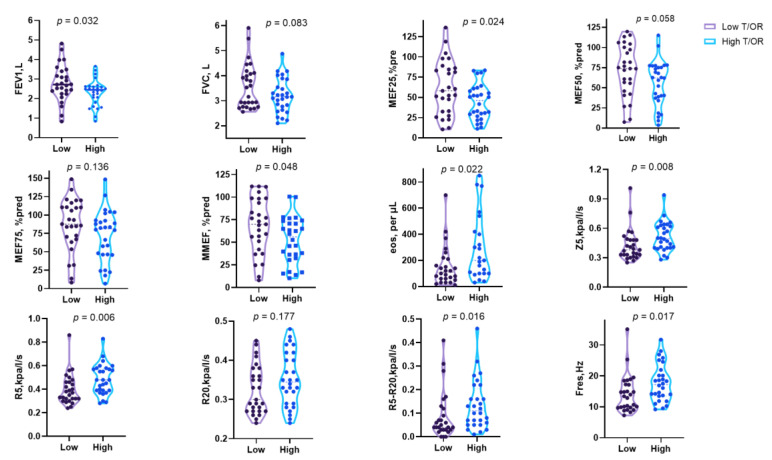
Spirometry and lung airway resistance parameters according to the airway wall thickness stratified by T/OR ratio. Data expressed as median and interquartile range.

Despite the underlying inflammation, airway obstruction and narrowing of the airway lumen are easily recognised through pulmonary function tests and chest image analysis. Indeed, there is a subgroup of asthma with irreversible or fixed airflow obstruction (FAO). This subtype is often underestimated since standard treatments such as inhaled corticosteroids and bronchodilators are likely to prevent asthma exacerbation, resulting in disease progression, a decline in lung function, and long-term mortality [21,22]. However, the clinical research data on this type of asthma are relatively scarce and still need to be investigated. Whether the particular cohort of patients insensitive to budesonide/formoterol in our study represents one subtype of FAO is unclear. However, the medication response may be explained from another perspective by the airway obstruction that could be assessed directly using the pulmonary function test. To verify whether baseline lung function contributed to the diverse drug response, we further compared the spirometry and IOS parameters in patients stratified by WA% and T/OR. As expected, we found that patients with worse responses to budesonide/formoterol had increased baseline FEV1 and MEF_25_ (Figure 5 and Figure 6). It makes sense because patients with baseline lung function preserved have limited space to reach the ceiling after bronchodilators, but the underlying inflammation and remodelling process is still ongoing. Furthermore, a study found that patients with a greater degree of mucus plugs and worse airway obstruction, measured by ventilation defect percentage, could benefit more from benralizumab therapy. This biologic targets eosinophils and, on the other hand, highlights the need to understand the underlying mechanisms [23]. In our study, we found that the baseline eosinophils are significantly increased in patients with high WA% and T/OR, which may serve as valuable biomarkers for asthma management (Figure 5 and Figure 6).

Phenotyping asthma is an essential step before starting medication, and a precise classification based on airway wall indicators may help to make precise medication strategies. It means that interventions targeting airway inflammation or airway remodelling may produce better outcomes than interventions targeting airway reversibility in patients with increased airway thickness. As the SMART therapy recommended by GINA reports [2], taking budesonide/formoterol as maintenance plus remission treatment is therefore an alternative. In this regard, patients with low WA% and/or T/OR may benefit from once daily administration, plus on-demand use of budesonide/formoterol to alleviate asthma inflammation, which can reduce the use of β2 receptor agonists, as the latter has limited effect on bronchodilation in patients with fixed obstruction [24,25].

In this study, we applied an artificial intelligence program to quantify airway metrics in patients with asthma. Since manual measurement of airways by HRCT scanning is time-consuming and error prone, the Airway Inspector module of the Chest Imaging Platform makes the results more objective and standardised, avoiding errors in manual measurement. However, this application can only provide transverse CT measurements in which the visible airways are required to be perpendicular to the vertical axis. Some small airways and non-vertical bronchi are thus neglected [26], although others have claimed to measure only five layers: the superior margin of the aortic arch, tracheal carina, one centimetre below the carina, inferior pulmonary veins, and two centimetres above the diaphragm [27]. As the proximal and distal airways present different characteristics, we disagree with such a method. Hence, in this study, all the visible bronchi were embraced to make the results more objective and representative. 

Our results suggest that the airway walls of patients with asthma are significantly thicker than those of healthy controls. In addition, it has been shown that higher airway wall thickness is associated with more severe disease in asthma. The results are comparable to other studies [11,28,29]. Benlala et al. [12] investigated the bronchial wall thickness using magnetic resonance imaging in which WA% was also proved higher in severe than in non-severe asthma patients. However, in our study, the absolute value of airway wall thickness in moderate to severe asthma made no difference compared to the healthy controls and mild asthma. It is reasonable because the bronchial tree comprised twenty-three generations [30]. The absolute value of wall thickness is affected mainly by the location of the airway measured. The quantitative CT method generally captures the proximal bronchial wall dimensions. Even at high resolution, CT does not permit direct visualisation of small airways (<2 mm in diameter) that comprise mainly the final eight divisions of the airways with respiratory bronchioles, alveolar ducts, and alveolar sacs [30]. To avoid this drawback, we used normalised values such as WA% and T/OR ratio that can effectively represent the overall degree of airway wall thickness. 

IOS reflects the respiratory system’s viscous and inertial/elastic properties by resistance (Rrs) and reactance (Xrs), providing more information on airway structural and functional characteristics [31]. In the present study, the airway resistance and reactance indicators function quite well as the spirometry in evaluating the asthma severity and bronchial structural changes. The bronchial wall thickness indicator (T/OR and WA%) correlated well with the lung function measured by plethysmography and impulse oscillometry [32]. However, the large airway resistance R20 did not correlate with the spirometry parameters, showing its advantage in assessing the pathological changes of airway obstruction and gas trapping. In the present study, IOS resistance parameters, including Z5, R5, and R5–R20, increased in patients with severe asthma; however, R20 made no difference between groups, suggesting that the increase in total resistance is exclusively due to resistance from the peripheral airways. In addition, the reactance indices X5 and Fres significantly increase in moderate to severe asthma patients compared to those with mild disease. It is because the Fres is a sensitive indicator to airway obstruction, at which point the forces of inertia and capacitance are equal [10,33]. Likewise, the reactance indices X5 reflect the physical properties of the lung parenchyma and its ability to expand and facilitate alveolar filling in peripheral airways [34].

We acknowledge some potential limitations that deserve comment. Firstly, our study did not assess the effect of previous treatment regimens on airway inflammation, which may have implications for studying airway wall metrics and drug responsiveness. Secondly, it is a single-centre study, and the population was comparatively small. However, it is worth mentioning that all enrolled patients could complete a twelve-hour lung function follow-up, which is very meaningful for us to dynamically observe lung function changes at different airway patterns. Thirdly, smoking and asthma evolution may have an impact on the bronchial wall thickness. Although we did not find an association between smoking status and airway characteristics, prospective studies with larger sample sizes may yield significant findings. In addition, bronchiectasis is common in asthma, especially in severe asthma, and our study excluded this portion of patients, and we hope to do more in-depth research on this topic in our future work.

## 5. Conclusions

Asthma patients with different bronchial wall thicknesses exhibited variable lung function changes. Specifically, patients with thick airway wall patterns were more sensitive to inhaled budesonide in the short term. Since chest HRCT metrics were well reflected by spirometry and IOS parameters, regularly monitoring spirometry and IOS may facilitate the follow-up and proper management of asthma.

## Figures and Tables

**Table 1:** Characteristics of asthma patients stratified by disease severity.

	Total *n* = 55	Mild Asthma *n* = 37 (67%)	Mod-Severe Asthma *n* = 18 (33%)	*p*-Value
Age, yrs	43.4 (12.2)	40.3 (10.6)	49.7 (13.1)	0.006
Gender (female)	30 (55%)	15 (41%)	15 (83%)	0.003
BMI (kg·m^−2^)	21.7 (20.2–24.6)	21.7 (20.2–24.79)	21.8 (21.0–23.8)	0.98
Smoking status				0.045
Non-smoker	45 (82%)	33 (89%)	12 (67%)	
Active smoker	8 (15%)	4 (11%)	4 (22%)	
Extra smoker	2 (4%)	0 (0%)	2 (11%)	
Blood EOS (μL^−1^)	140 (70–290)	100 (60–210)	290 (150–420)	0.015
Blood EOS (%)	2.25 (1.20–4.30)	2.00 (1.05–3.70)	4.25 (3.00–6.20)	0.015
	Impulse oscillometry			
Rc, kpa/L/s	0.25 (0.13–0.28)	0.25 (0.13–0.26)	0.26 (0.23–0.32)	0.075
Rp, kpa/L/s	0.25 (0.20–0.35)	0.25 (0.15–0.25)	0.40 (0.25–0.55)	<0.001
Z5, kpa/L/s	0.43 (0.34–0.57)	0.40 (0.33–0.49)	0.58 (0.42–0.67)	0.001
R5, kpa/L/s	0.42 (0.33–0.55)	0.39 (0.32–0.48)	0.54 (0.39–0.58)	0.004
R20, kpa/L/s	0.33 (0.28–0.39)	0.33 (0.28–0.39)	0.34 (0.28–0.37)	0.90
R5-R20, kpa/L/s	0.07 (0.04–0.16)	0.06 (0.03–0.10)	0.17 (0.09–0.27)	<0.001
X5, kpa/L/s	−0.11 (−0.16–0.08)	−0.10 (−0.11–0.08)	−0.21 (−0.29–0.14)	<0.001
Fres, Hz	14.75 (10.35–18.87)	13.65 (10.07–16.07)	19.48 (17.20–25.28)	<0.001
	Lung Function Parameters			
FEV1, L	2.5 (2.07–3.07)	2.6 (2.4–3.3)	1.7 (1.5–2.5)	<0.001
FVC, L	3.2 (2.76–4.0)	3.23 (2.92–4.07)	3.0 (2.6–3.8)	0.049
FEV1, %pred	86.8 (73.3–99.5)	97.2 (86.8–104.1)	63.8 (51.9–73.3)	<0.001
FVC, %pred	93.5 (83.1–104.7)	100.8 (88.8–110.4)	83.7 (73.2–91.5)	<0.001
FEV1/FVC	77.7 (67.7–84.0)	81.6 (76.8–86.1)	60.7 (46.9–69.0)	<0.001
RV, %pred	109.1 (38.9)	95.97 (21.6)	136.2 (51.6)	<0.001
TLC, %pred	96.8 (11.2)	95.98 (8.8)	98.6 (15.1)	0.42
RV/TLC	35.6 (10.5)	31.02 (5.0)	45.03 (12.6)	<0.001
MEF75, %pred	79.2 (35.7)	98.56 (21.7)	39.24 (23.0)	<0.001
MEF50, %pred	63.5 (30.7)	80.17 (20.2)	29.13 (16.6)	<0.001
MEF25, %pred	53.1 (29.4)	67.30 (24.9)	24.0 (10.4)	<0.001
MMEF, %pred	60.0 (29.1)	75.68 (20.1)	27.8 (14.2)	<0.001
PEF, %pred	91.3 (26.2)	105.2 (15.3)	62.8 (20.2)	<0.001

Rc, central resistance; Rp, peripheral resistance; Z5, the respiratory impedance; R5 and R20, respiratory system resistance at 5 and 20 Hz, respectively; X5, respiratory system reactance at 5 Hz; Fres, resonant frequency; FEV1, forced expiratory volume in one second; FVC, forced vital capacity; RV, residual volume; TLC, total lung capacity; MEF, maximal expiratory flow; MMEF, maximal mid-expiratory flow; PEF, peak expiratory flow.

**Table 2:** Computed tomography (CT) metrics in asthma and heathy controls.

	Control *n* = 51	Asthma *n* = 55	*p*-Value	Mild Asthma *n* = 37	Mod-Severe Asthma *n* = 18	*p*-Value
Age, yrs	40.5 (11.3)	43.4 (12.2)	0.2	40.3 (10.6)	49.7 (13.1)	0.006
Gender, female	28 (54.9%)	30 (55%)	0.97	15 (41%)	15 (83%)	0.003
No. of bronchi	15.5 (12.0–19.0)	19.0 (15.0–24.5)	0.002	19.0 (16.5–23.5)	20.3 (14.5–25.5)	0.96
IR, mm	2.07 (1.93–2.38)	1.78 (1.57–2.09)	<0.001	1.91 (1.63–2.13)	1.70 (1.51–1.85)	0.055
OR, mm	3.97 (3.70–4.18)	3.63 (3.40–3.88)	<0.001	3.70 (3.53–3.90)	3.55 (3.39–3.64)	0.082
Ai (mm^2^)	15.38 (13.79–20.08)	11.62 (9.31–15.67)	<0.001	13.14 (9.55–16.86)	11.01 (9.09–12.92)	0.095
Ao (mm^2^)	52.55 (46.35–60.35)	44.86 (39.47–51.08)	<0.001	45.89 (41.80–52.49)	42.38 (39.47–45.24)	0.13
WT, mm	1.76 (1.68–1.93)	1.78 (1.70–1.88)	0.93	1.77 (1.68–1.87)	1.79 (1.75–1.88)	0.11
T/OR	0.46 (0.43–0.48)	0.50 (0.47–0.54)	<0.001	0.48 (0.46–0.52)	0.51 (0.50–0.55)	0.029
WA%	71.21 (68.35–74.06)	75.53 (72.51–78.68)	<0.001	73.65 (71.46–77.58)	76.78 (74.86–79.66)	0.023
Pi, mm	13.00 (12.10–14.93)	11.20 (9.85–13.14)	<0.001	11.98 (10.21–13.36)	10.67 (9.49–11.65)	0.055
Pi10			<0.001			0.51
<10 mm	14 (25%)	1 (2%)		8 (22%)	6 (33%)	
>10 mm	41 (75%)	50 (98%)		29 (78%)	12 (67%)	
Sqrt (WA) (mm)	5.97 (5.61–6.17)	5.63 (5.35–5.83)	<0.001	5.64 (5.36–5.83)	5.53 (5.30–5.83)	0.58

Internal radius (IR); Outer radius (OR), Wall thickness (WT); Ratio of WT and OR (T/OR); Wall area percentage (WA%); Perimeter of 10 mm (Pi10); Square root of wall area (Sqrt WA); Luminal area (Ai); Cross-sectional area (Ao).

## Data Availability

The data and/or related materials of this study are available from the corresponding author on reasonable request.

## Supplementary Materials

The following supporting information can be downloaded at https://www.mdpi.com/article/10.3390/jcm12020639/s1, Figure S1: Correlation between T/OR and lung function measured by spirometry and IOS. Figure S2: Temporal changes of lung function based on T/OR. Table S1: Correlation between the index of wall thickness and lung function parameters in healthy controls.

## Abbreviations

ACQ	asthma control questionnaire
Ai	luminal area
Ao	cross-sectional area of airway
COPD	chronic obstructive pulmonary disease
FEV1	forced expiratory volume in one second
Fres	resonant frequency
FVC	forced vital capacity
HRCT	high-resolution computed tomography
IOS	impulse oscillometry
IQR	interquartile range
MEF75	maximal expiratory flow at 75% of the FVC
MEF50	maximal expiratory flow at 50% of the FVC
MEF25	maximal expiratory flow at 25% of the FVC
MMEF	maximal mid-expiratory flow
PEF	peak expiratory flow
Pi10	inner airway perimeter of 10 mm
Rc	central resistance
Rp	peripheral resistance
RV	residual volume
R5	resistance at 5 Hz
R20	resistance at 20 Hz
TLC	total lung capacity
T/OR	ratio of airway wall thickness to the outer radius
WT	wall thickness
WA%	wall area percentage
X5	reactance at 5 Hz
Z5	impedance value at 5 Hz
