# Supplementary material for: Quantitative CT Metrics for the Prediction of Therapeutic Effect in Asthma
Source: J Clin Med. 2023 Jan 13;12(2):639. doi: 10.3390/jcm12020639 (PMC9861330; doi:10.3390/jcm12020639)
Supplement: Supplementary file 1 [file jcm-12-00639-s001.zip › jcm-2116223-supplementary.pdf]

Table S1. Correlation between index of the wall thickness and lung function parameters in healthy controls.

	WA%		T/OR	
Variable	Rho	*p*-value	Rho	*p*-value
FEV1	−0.1908	ns	−0.1909	ns
FVC	−0.2108	ns	−0.1997	ns
FEV1/FVC	0.0269	ns	−0.0191	ns
MEF75	−0.2764	<0.05	−0.2849	<0.05
MEF50	−0.1352	ns	−0.1634	ns
MEF25	−0.1322	ns	−0.1576	ns
MMEF	−0.1412	ns	−0.1684	ns
PEF	−0.2582	ns	−0.2547	ns
Rp	0.1987	ns	0.2106	ns
R5	0.2557	ns	0.2623	ns
R20	0.1832	ns	0.1998	ns
R5-R20	0.2124	ns	0.1966	ns
Z5	0.2539	ns	0.2593	ns
X5	−0.1543	ns	−0.1712	ns
Fres	0.2704	ns	0.2699	ns

ns, no significant.

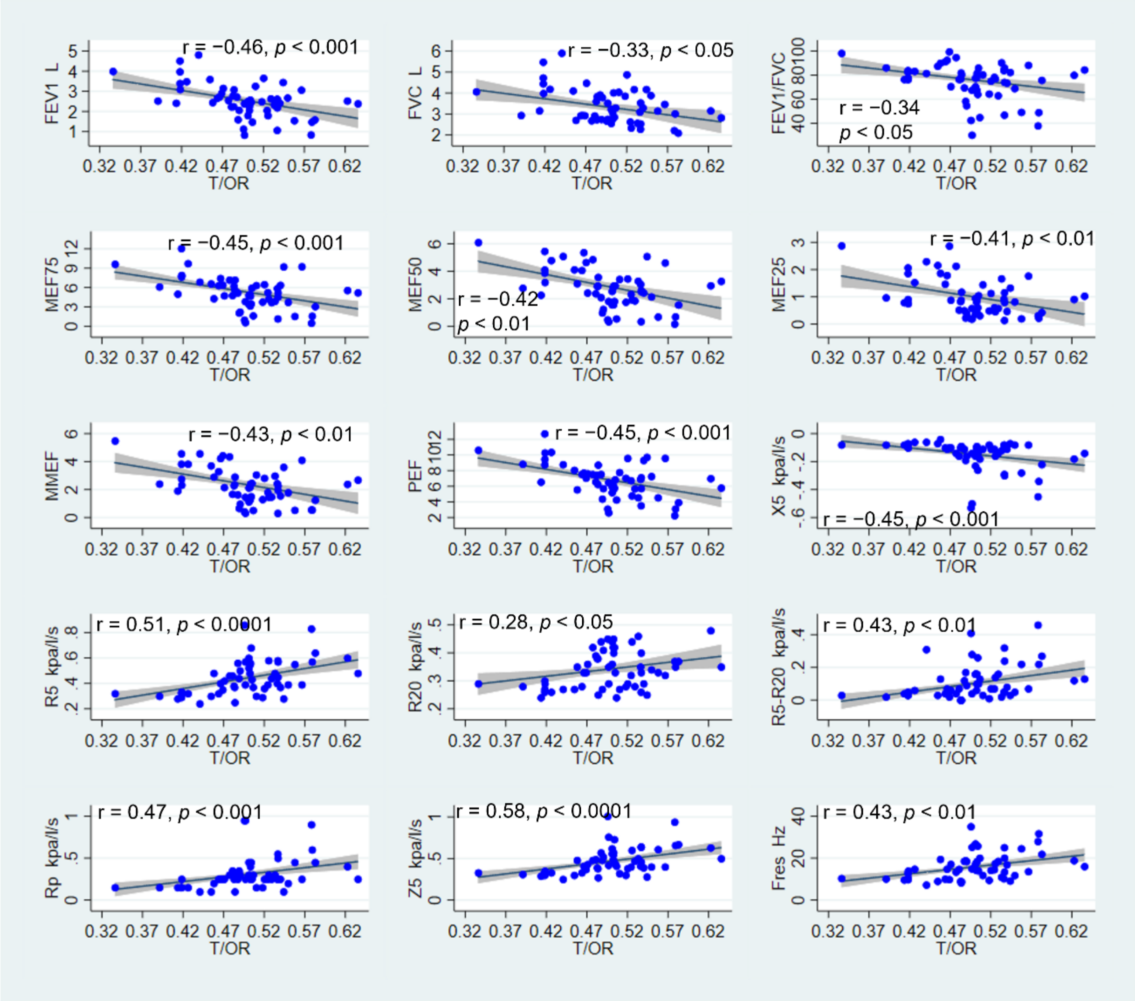

Figure S1. Correlation between T/OR and lung function measured by spirometry and IOS.

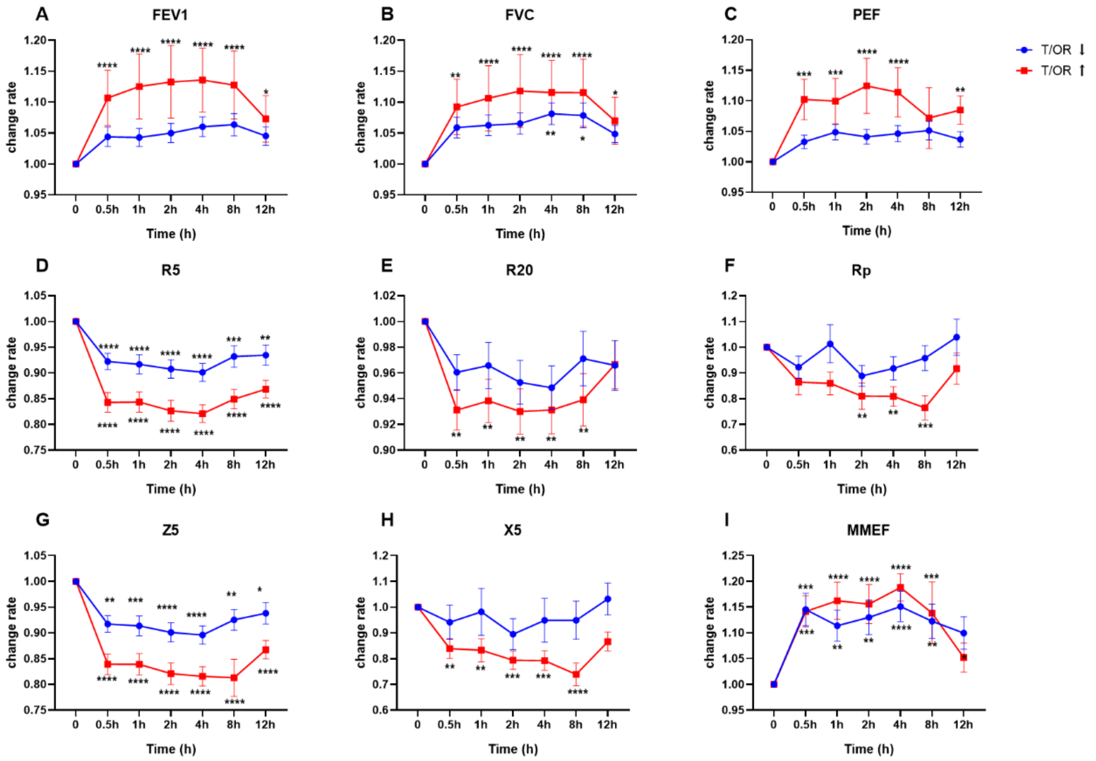

Figure S2. Temporal changes of lung function based on T/OR. (**A**) FEV1, forced expiratory volume in one second; (**B**) FVC, forced vital capacity; (**C**) PEF, peak expiratory flow; (**D**) R5, airway resistance at 5Hz; (**E**) R20, airway resistance at 20Hz; (**F**) Rp, peripheral resistance; (**G**) Z5, impedance value at 5 Hz; (**H**) X5, reactance at 5Hz; (**I**) MMEF, maximal mid-expiratory flow. The blue symbol means subject with relative non-thickened airways, and the red colour represents cohort with relative thickened airways. The dichotomisation threshold was generated based on the median value of T/OR. * $p < 0.05$, ** $p < 0.01$, *** $p < 0.001$, **** $p < 0.0001$. Comparisons were made between the specific timepoint and baseline lung function.
